# The Hospital Kitchen

**Published:** 1907-07-20

**Authors:** R. Langton Cole

**Affiliations:** 23 Throgmorton Street, E.C.


### Editor's Letter-Box.

[Our Correspondents are reminded that prolixity is a great bar to publication, and that brevity of style and conciseness of statement greatly facilitate early insertion.]

### THE HOSPITAL KITCHEN.

Sir,—In your valuable article on "The Hospital Kitchen," of July 6 last, there appears a statement which should not, I think, go uncorrected. In describing a trolley to be placed under the vegetable sinks you say: "Through this the water flows into the soil pipe." Now the words "soil pipe" have a perfectly definite meaning in sanitary work, and refer to a pipe taking excremental matter from a w.c. or slop sink. I do not suppose that anyone concerned with hospitals would connect the waste from a sink to a soil pipe, at any rate without some intervening trap; but the words in your article certainly suggest some such arrangement. I presume what is intended is, that the water should flow from the perforated tube on to a floor channel, and thence through a gully to the drains, or through a floor trap to a vertical waste pipe, if the kitchen is on an upper floor.

In the same article the word "boilers" is used for two quite different things, and I think this might be made clear.

Yours faithfully,

R. Langton Cole, F.R.I.B.A.

23 Throgmorton Street, E.C., July 12, 1907.

∴ The trolley placed under the vegetable sink does not take the place of an efficient trap, but is an additional precaution to prevent the mixture of grease and sand, as is pointed out in the article. Some manufacturers use the term "steam pan," and others "steam boiler" for the same fitting.—Ed., The Hospital.

THE HOSPITAL *July* 20, 1907.

